# (±)-3-Carb­oxy-2-(imidazol-3-ium-1-yl)­propanoate

**DOI:** 10.1107/S1600536809018510

**Published:** 2009-05-29

**Authors:** Sarah A. Reeb, Marlesa C. Shields, Kraig A. Wheeler

**Affiliations:** a Eastern Illinois University, Department of Chemistry, 600 Lincoln Avenue, Charleston, IL 61920-3099, USA

## Abstract

The title compound, C_7_H_8_N_2_O_4_, crystallizes as a zwitterion, with mol­ecules organized into mol­ecular sheets *via* carbox­yl–carboxyl­ate and N^+^—H⋯carboxyl­ate contacts. These sheets are constructed from translationally related mol­ecules that further link to neighboring motifs *via* π-stacking [centroid–centroid distance 3.504 (3) Å] and weak C—H⋯O contacts.

## Related literature

For related compounds, see: Centnerzwer (1899 ▶); Pasteur (1853 ▶); Wheeler *et al.* (2008 ▶). For a description of the Cambridge Structural Database, see: Allen (2002 ▶).
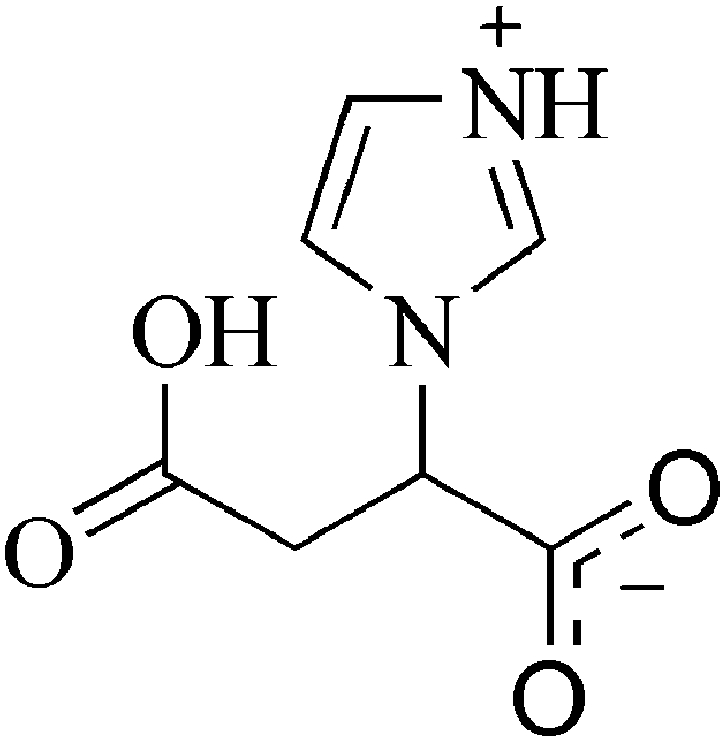


## Experimental

### 

#### Crystal data

C_7_H_8_N_2_O_4_
*M* *_r_* = 184.15
Monoclinic, 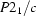

*a* = 7.6328 (7) Å
*b* = 7.4701 (7) Å
*c* = 13.7616 (12) Å
β = 96.752 (1)°
*V* = 779.21 (12) Å^3^
*Z* = 4
Mo *K*α radiation
μ = 0.13 mm^−1^
*T* = 296 K
0.38 × 0.28 × 0.18 mm

#### Data collection

Bruker *P*4 CCD diffractometer
Absorption correction: multi-scan (*SADABS*; Bruker, 2008 ▶) *T*_min_ = 0.952, *T*_max_ = 0.977
4668 measured reflections
1540 independent reflections
1254 reflections with *I* > 2σ(*I*)
*R*_int_ = 0.021

#### Refinement

*R*[*F* ^2^ > 2σ(*F* ^2^)] = 0.042
*wR*(*F* ^2^) = 0.115
*S* = 1.06
1540 reflections
126 parameters
H atoms treated by a mixture of independent and constrained refinement
Δρ_max_ = 0.24 e Å^−3^
Δρ_min_ = −0.26 e Å^−3^

### 

Data collection: *APEX2* (Bruker, 2008 ▶); cell refinement: *SAINT* (Bruker, 2008 ▶); data reduction: *SAINT* and *XPREP* (Bruker, 2008 ▶); program(s) used to solve structure: *SHELXS97* (Sheldrick, 2008 ▶); program(s) used to refine structure: *SHELXL97* (Sheldrick, 2008 ▶); molecular graphics: *X-SEED* (Barbour, 2001 ▶); software used to prepare material for publication: *X-SEED*.

## Supplementary Material

Crystal structure: contains datablocks global, I. DOI: 10.1107/S1600536809018510/hk2685sup1.cif

Structure factors: contains datablocks I. DOI: 10.1107/S1600536809018510/hk2685Isup2.hkl

Additional supplementary materials:  crystallographic information; 3D view; checkCIF report

## Figures and Tables

**Table 1:** Hydrogen-bond geometry (Å, °)

*D*—H⋯*A*	*D*—H	H⋯*A*	*D*⋯*A*	*D*—H⋯*A*
O3—H1⋯O2^i^	0.95 (3)	1.62 (3)	2.5440 (18)	163 (3)
N2—H3⋯O1^ii^	0.89 (3)	1.85 (3)	2.732 (2)	170 (2)
C7—H7⋯O1^iii^	0.93	2.42	3.333 (2)	168
C5—H5⋯O4^iv^	0.93	2.57	3.260 (3)	131

Symmetry codes: (i) 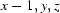; (ii) 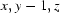; (iii) 
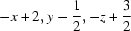
; (iv) 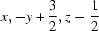.

**Fig. 1.**
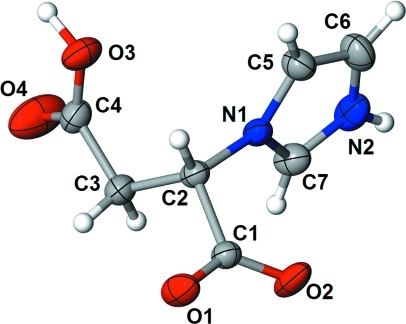
The molecular structure of the title molecule, with the atom-numbering scheme. Displacement ellipsoids are drawn at the 50% probability level.

**Fig. 2.**
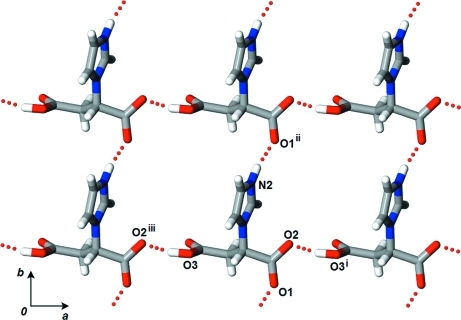
A partial packing diagram of the title compound, showing molecular sheets constructed from carboxyl···carboxylate and N—H···O contacts [symmetry codes: (i) 1 + x, y, z, (ii) x, 1 + y, z; (iii) x - 1, y, z].

**Fig. 3.**
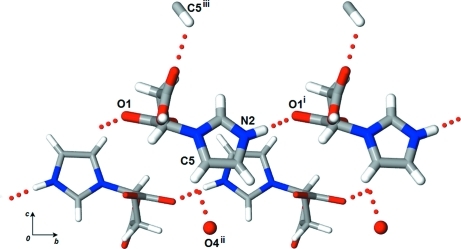
Projection showing alignment of molecular sheets with imidazolium π stacking and C—H···O interactions [symmetry codes: i) x, y - 1, z, (ii) x, 3/2 - y, 1/2 + z; (iii) x - 3, 3/2 - y, z - 1/2].

**Fig. 4.**
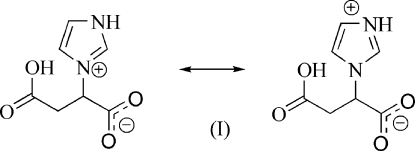
The tautomeric forms of the title compound.

## supplementary crystallographic information

### Comment

Our recent reinvestigation of Pasteur's 1853 quasiracemates (Pasteur, 1853; Wheeler *et al.*, 2008) has motivated us to explore other examples of these unusual materials of historical and supramolecular importance. In 1899, Centnerzwer reported that mixtures of (+)-chlorosuccinic acid and (-)-bromosuccinic acid formed a binary compound that also exhibited quasiracemic behavior (Centnerzwer, 1899). Our initial attempts to grow crystals of this quasiracemic phase were unsuccessful. This result was somewhat anticipated given that Centnerzwer's melting point phase diagrams showed the crystal stabilities of the homochiral phases more stable than the quasiracemate. We then turned our attention to investigating the effects of co-crystalline additives to crystal growth of this quasiracemate and the corresponding racemic and homochiral compounds. During the course of these co-crystal screening investigations, we observed the formation of crystals of the title compound from slow evaporation of a methanol:CH_2_Cl_2_ (1:1) solution of (±)-2-chlorosuccinic acid and imidazole.

The title compound, (I), formed from the substitution reaction of imidazole and 2-chlorosuccinic acid, crystallizes in space group *P*2_1_/*c* as the imidazolium carboxylate zwitterion (Fig. 1). Inspection of the molecular structure reveals a resonance stabilized imidazolium ring with (N1—C7) - (N2—C7) = +0.038 Å. A search of the Cambridge Structural Database (CSD, Version 5.30 with August 2008 and February 2009 updates; Allen, 2002) for other *N*-alkylimidazolium fragments uncovered 44 organic structures. This collection shows similar bonding patterns to (I) with a concentration of Δ(N—C) values near 0.00, +0.01, and +0.03 Å.

The crystal structure of (I) is organized by a complex blend of strong and weak intermolecular contacts (Table 1). Neighboring molecules are linked by carboxyl···carboxylate interactions to give a catemeric motif that propagates along the a-axis (Fig. 2). This motif is extended by N2^+^—*H*···carboxylate contacts to produce a molecular sheet in the *ab* plane. The participation of the imidazolium N^+^—H group in hydrogen bonding is also a common feature in the 44 structures retrieved from the CSD. Each of these structures show N^+^—H···A contacts with a diverse set of acceptors [A = oxygen(52), nitrogen(3), halogen(15), or π(2); 72 contacts]. Interestingly, each molecular sheet in (I) consists of translationally related molecules with imidazolium groups exposed on one side of the motif and carboxyl O4 atoms on the other side. The crystal structure of (I) is characterized by the stacking of these molecular sheets with adjacent motifs related by inversion symmetry and linked by either interdigitated imadazolium···imidazolium stacks [3.504 (3) Å] or weak C5—H5···O4 interactions (Fig. 3).

### Experimental

Single crystals of the title compound were prepared by slow evaporation at room temperature of a methanol:CH_2_Cl_2_ (1:1) solution of (±)-2-chlorosuccinic acid and imidazole (1:1).

### Refinement

H atoms (for OH and NH) were located in difference Fourier synthesis and refined isotropically. The remaining H atoms were positioned geometrically with C—H = 0.93, 0.98 and 0.97 Å, for aromatic, methine and metnylene H atoms, respectively, and constrained to ride on their parent atoms, with U_iso_(H) = 1.2U_eq_(C).

### Crystal data

**Table:**

C_7_H_8_N_2_O_4_	*F*(000) = 384
*M**_r_* = 184.15	*D*_x_ = 1.570 Mg m^−^^3^
Monoclinic, *P*2_1_/*c*	Mo *K*α radiation, λ = 0.71073 Å
Hall symbol: -P 2ybc	Cell parameters from 1902 reflections
*a* = 7.6328 (7) Å	θ = 6.0–54.6°
*b* = 7.4701 (7) Å	µ = 0.13 mm^−^^1^
*c* = 13.7616 (12) Å	*T* = 296 K
β = 96.752 (1)°	Transparent prism, colourless
*V* = 779.21 (12) Å^3^	0.38 × 0.28 × 0.18 mm
*Z* = 4	

### Data collection

**Table:**

Bruker P4 CCD diffractometer	1540 independent reflections
Radiation source: fine-focus sealed tube	1254 reflections with *I* > 2σ(*I*)
graphite	*R*_int_ = 0.021
φ and ω scans	θ_max_ = 26.0°, θ_min_ = 2.7°
Absorption correction: multi-scan (*SADABS*; Bruker, 2008)	*h* = −9→9
*T*_min_ = 0.952, *T*_max_ = 0.977	*k* = −9→8
4668 measured reflections	*l* = −16→16

### Refinement

**Table:**

Refinement on *F*^2^	Primary atom site location: structure-invariant direct methods
Least-squares matrix: full	Secondary atom site location: difference Fourier map
*R*[*F*^2^ > 2σ(*F*^2^)] = 0.042	Hydrogen site location: inferred from neighbouring sites
*wR*(*F*^2^) = 0.115	H atoms treated by a mixture of independent and constrained refinement
*S* = 1.06	*w* = 1/[σ^2^(*F*_o_^2^) + (0.0548*P*)^2^ + 0.3288*P*] where *P* = (*F*_o_^2^ + 2*F*_c_^2^)/3
1540 reflections	(Δ/σ)_max_ < 0.001
126 parameters	Δρ_max_ = 0.24 e Å^−^^3^
0 restraints	Δρ_min_ = −0.26 e Å^−^^3^

### Special details

**Table:**

Experimental. The instrument used for data collection was a Bruker P4 with a APEXII CCD detector upgrade.
Geometry. All e.s.d.'s (except the e.s.d. in the dihedral angle between two l.s. planes) are estimated using the full covariance matrix. The cell e.s.d.'s are taken into account individually in the estimation of e.s.d.'s in distances, angles and torsion angles; correlations between e.s.d.'s in cell parameters are only used when they are defined by crystal symmetry. An approximate (isotropic) treatment of cell e.s.d.'s is used for estimating e.s.d.'s involving l.s. planes.
Refinement. Refinement of *F*^2^ against ALL reflections. The weighted *R*-factor *wR* and goodness of fit *S* are based on *F*^2^, conventional *R*-factors *R* are based on *F*, with *F* set to zero for negative *F*^2^. The threshold expression of *F*^2^ > σ(*F*^2^) is used only for calculating *R*-factors(gt) *etc*. and is not relevant to the choice of reflections for refinement. *R*-factors based on *F*^2^ are statistically about twice as large as those based on *F*, and *R*-factors based on ALL data will be even larger.

### Fractional atomic coordinates and isotropic or equivalent isotropic displacement parameters (Å^2^)

**Table:**

	*x*	*y*	*z*	*U*_iso_*/*U*_eq_	
O1	1.00100 (17)	1.13604 (17)	0.63749 (11)	0.0446 (4)	
O2	1.09600 (17)	0.86066 (19)	0.61600 (13)	0.0558 (5)	
O3	0.42339 (18)	0.9309 (2)	0.62306 (10)	0.0475 (4)	
O4	0.4539 (2)	0.8529 (3)	0.77836 (13)	0.0813 (7)	
N1	0.77182 (17)	0.72061 (19)	0.57875 (10)	0.0293 (3)	
N2	0.8241 (2)	0.4386 (2)	0.57164 (14)	0.0448 (4)	
C1	0.9788 (2)	0.9740 (2)	0.62128 (12)	0.0296 (4)	
C2	0.7853 (2)	0.9081 (2)	0.61136 (12)	0.0290 (4)	
H2	0.7163	0.9819	0.5619	0.035*	
C3	0.7111 (2)	0.9343 (3)	0.70828 (13)	0.0367 (4)	
H3A	0.7738	0.8551	0.7563	0.044*	
H3B	0.7355	1.0561	0.7301	0.044*	
C4	0.5163 (2)	0.9005 (3)	0.70683 (14)	0.0378 (4)	
C5	0.7043 (2)	0.6631 (3)	0.48706 (14)	0.0391 (5)	
H5	0.6466	0.7330	0.4373	0.047*	
C6	0.7375 (3)	0.4871 (3)	0.48305 (16)	0.0474 (5)	
H6	0.7071	0.4121	0.4298	0.057*	
C7	0.8438 (2)	0.5806 (2)	0.62777 (15)	0.0400 (5)	
H7	0.8993	0.5830	0.6917	0.048*	
H1	0.301 (5)	0.920 (4)	0.630 (2)	0.098 (10)*	
H3	0.870 (3)	0.333 (4)	0.5910 (17)	0.061 (7)*	

### Atomic displacement parameters (Å^2^)

**Table:**

	*U*^11^	*U*^22^	*U*^33^	*U*^12^	*U*^13^	*U*^23^
O1	0.0352 (7)	0.0277 (7)	0.0694 (10)	−0.0049 (5)	−0.0004 (6)	−0.0018 (6)
O2	0.0231 (7)	0.0362 (8)	0.1069 (13)	0.0015 (6)	0.0032 (7)	−0.0128 (8)
O3	0.0277 (7)	0.0630 (10)	0.0517 (9)	−0.0032 (6)	0.0042 (6)	0.0051 (7)
O4	0.0476 (10)	0.1370 (19)	0.0607 (11)	−0.0101 (11)	0.0125 (8)	0.0310 (11)
N1	0.0244 (7)	0.0263 (8)	0.0370 (8)	−0.0015 (5)	0.0021 (6)	−0.0016 (6)
N2	0.0378 (9)	0.0255 (9)	0.0715 (12)	0.0012 (7)	0.0078 (8)	0.0026 (8)
C1	0.0255 (8)	0.0299 (9)	0.0330 (9)	−0.0015 (7)	0.0021 (6)	−0.0006 (7)
C2	0.0249 (8)	0.0259 (9)	0.0356 (9)	0.0001 (6)	0.0006 (6)	−0.0008 (7)
C3	0.0290 (9)	0.0422 (11)	0.0387 (10)	0.0019 (8)	0.0028 (7)	−0.0039 (8)
C4	0.0308 (9)	0.0384 (10)	0.0449 (11)	0.0021 (7)	0.0078 (8)	0.0001 (8)
C5	0.0409 (10)	0.0372 (11)	0.0383 (10)	−0.0025 (8)	0.0007 (8)	−0.0041 (8)
C6	0.0541 (13)	0.0360 (11)	0.0527 (13)	−0.0051 (9)	0.0079 (10)	−0.0120 (9)
C7	0.0374 (10)	0.0317 (10)	0.0492 (11)	−0.0007 (8)	−0.0023 (8)	0.0039 (8)

### Geometric parameters (Å, °)

**Table:**

O1—C1	1.239 (2)	C1—C2	1.548 (2)
O2—C1	1.240 (2)	C2—C3	1.522 (2)
O3—C4	1.300 (2)	C2—H2	0.9800
O3—H1	0.95 (3)	C3—C4	1.506 (3)
O4—C4	1.197 (2)	C3—H3A	0.9700
N1—C7	1.328 (2)	C3—H3B	0.9700
N1—C5	1.374 (2)	C5—C6	1.341 (3)
N1—C2	1.471 (2)	C5—H5	0.9300
N2—C7	1.310 (3)	C6—H6	0.9300
N2—C6	1.366 (3)	C7—H7	0.9300
N2—H3	0.89 (3)		
			
C4—O3—H1	110 (2)	C4—C3—H3A	108.3
C7—N1—C5	107.96 (15)	C2—C3—H3A	108.3
C7—N1—C2	125.82 (15)	C4—C3—H3B	108.3
C5—N1—C2	125.65 (15)	C2—C3—H3B	108.3
C7—N2—C6	108.73 (17)	H3A—C3—H3B	107.4
C7—N2—H3	121.9 (16)	O4—C4—O3	123.55 (18)
C6—N2—H3	129.2 (16)	O4—C4—C3	121.78 (18)
O1—C1—O2	126.38 (16)	O3—C4—C3	114.66 (16)
O1—C1—C2	115.82 (15)	C6—C5—N1	106.96 (18)
O2—C1—C2	117.74 (15)	C6—C5—H5	126.5
N1—C2—C3	111.71 (14)	N1—C5—H5	126.5
N1—C2—C1	111.19 (13)	C5—C6—N2	107.30 (18)
C3—C2—C1	109.36 (13)	C5—C6—H6	126.3
N1—C2—H2	108.2	N2—C6—H6	126.3
C3—C2—H2	108.2	N2—C7—N1	109.04 (17)
C1—C2—H2	108.2	N2—C7—H7	125.5
C4—C3—C2	115.82 (15)	N1—C7—H7	125.5
			
C7—N1—C2—C3	−59.2 (2)	C2—C3—C4—O4	152.5 (2)
C5—N1—C2—C3	130.59 (17)	C2—C3—C4—O3	−28.9 (2)
C7—N1—C2—C1	63.3 (2)	C7—N1—C5—C6	0.1 (2)
C5—N1—C2—C1	−106.93 (18)	C2—N1—C5—C6	171.80 (16)
O1—C1—C2—N1	172.03 (15)	N1—C5—C6—N2	0.1 (2)
O2—C1—C2—N1	−10.5 (2)	C7—N2—C6—C5	−0.3 (2)
O1—C1—C2—C3	−64.1 (2)	C6—N2—C7—N1	0.3 (2)
O2—C1—C2—C3	113.28 (18)	C5—N1—C7—N2	−0.3 (2)
N1—C2—C3—C4	−64.66 (19)	C2—N1—C7—N2	−171.95 (16)
C1—C2—C3—C4	171.82 (15)		

### Hydrogen-bond geometry (Å, °)

**Table:**

*D*—H···*A*	*D*—H	H···*A*	*D*···*A*	*D*—H···*A*
O3—H1···O2^i^	0.95 (3)	1.62 (3)	2.5440 (18)	163 (3)
N2—H3···O1^ii^	0.89 (3)	1.85 (3)	2.732 (2)	170 (2)
C7—H7···O1^iii^	0.93	2.42	3.333 (2)	168
C5—H5···O4^iv^	0.93	2.57	3.260 (3)	131

Symmetry codes: (i) *x*−1, *y*, *z*; (ii) *x*, *y*−1, *z*; (iii) −*x*+2, *y*−1/2, −*z*+3/2; (iv) *x*, −*y*+3/2, *z*−1/2.
